# The macular mapping test: a reliability study

**DOI:** 10.1186/1471-2415-5-18

**Published:** 2005-08-10

**Authors:** Hannah Bartlett, Leon N Davies, Frank Eperjesi

**Affiliations:** 1 Ophthalmic and Physiological Optics Research Group, Aston University, Birmingham, UK

## Abstract

**Background:**

Age-related macular degeneration (ARMD) is the leading cause of visual disability in people over 60 years of age in the developed world. The success of treatment deteriorates with increased latency of diagnosis. The purpose of this study was to determine the reliability of the macular mapping test (MMT), and to investigate its potential as a screening tool.

**Methods:**

The study population comprised of 31 healthy eyes of 31 participants. To assess reliability, four macular mapping test (MMT) measurements were taken in two sessions separated by one hour by two practitioners, with reversal of order in the second session. MMT readings were also taken from 17 age-related maculopathy (ARM), and 12 AMD affected eyes.

**Results:**

For the normal cohort, average MMT scores ranged from 85.5 to 100.0 MMT points. Scores ranged from 79.0 to 99.0 for the ARM group and from 9.0 to 92.0 for the AMD group. MMT scores were reliable to within ± 7.0 points. The difference between AMD affected eyes and controls (z = 3.761, p = < 0.001) was significant. The difference between ARM affected eyes and controls was not significant (z = -0.216, p = 0.829).

**Conclusion:**

The reliability data shows that a change of 14 points or more is required to indicate a clinically significant change. This value is required for use of the MMT as an outcome measure in clinical trials. Although there was no difference between MMT scores from ARM affected eyes and controls, the MMT has the advantage over the Amsler grid in that it uses a letter target, has a peripheral fixation aid, and it provides a numerical score. This score could be beneficial in office and home monitoring of AMD progression, as well as an outcome measure in clinical research.

## Background

Age-related macular degeneration (AMD) is the advanced from of age-related macular disease (ARMD) and is the leading cause of visual disability in patients over the age of 60 years in the developed World [1]. In the United States, the demographic right shift is expected to cause an increase in the number of people affected by this condition from 1.75 million to almost 3 million by 2020 [2].

Exudative AMD and/or geographic atrophy (GA) can result in the severe visual loss, and their prevalence in the US population over 40 years of age has been estimated at 1.47% [95% confidence interval (CI), 1.38% – 1.55%] [2]. The likelihood of visual deterioration in those with exudative AMD may be reduced with laser photocoagulation and photodynamic therapy [3-6], but the success rate deteriorates with increasing latency of diagnosis as lesions extend towards the foveal avascular zone. Consequently, early diagnosis is crucial, and at risk patients are often advised to use an Amsler grid at home for detection of scotomas and metamorphopsia [7-9].

The Amsler grid has been used in clinical practice since the 1940s [10] for detecting and monitoring retinal conditions such as age-related macular disease [11-13]. It has been shown, however, that a report of distortion of the Amsler grid can come from perceived lines filling-in across scotomas, or equally from non-scotomatous retinal impairments; the clinician has no way of knowing which. Seventy-seven percent of standard and 87 % of threshold scotomas that are 6° or less in diameter are not detected by Amsler grid testing [10]. The poor performance of the Amsler grid may be related to the inability to maintain fixation while testing the peripheral visual field [14], crowding effects caused by peripheral presentation of multiple lines [15], inability to assess factors such as quality of examination performance, and low compliance to perform the Amsler grid at home [14]. Despite the shortcomings of this test, the Amsler has been used as a measure of visual change in a randomised controlled trial (RCT) investigating the effect of nutritional supplementation in atrophic AMD [16].

The MMT was designed primarily for quick assessment of residual vision in patients with maculopathies. However, the peripheral fixation aid, along with the use of letter targets may make it a useful tool for monitoring progression of macular disease. The objective of this study was to assess reliability (repeatability and reproducibility), and to assess the effect of ARMD on MMT scores.

The results of this study will allow the use of the MMT as an outcome measure in clinical research, and may also be of interest to those who evaluate scotomas for the purpose of eccentric viewing training.

## Methods

### Design and setting

This was a reliability study designed to investigate the reproducibility and repeatability of the MMT. Data was also collected from ARMD affected eyes to assess the impact of the disease on MMT score. The study was carried out in the Neurosciences Research Institute at Aston University, Birmingham, UK, in a clinical research setting.

### Subjects

Thirty-one normally sighted participants were recruited from staff, students, and patients of the Division of Optometry, Aston University, Birmingham, UK. This research adhered to the tenets of the Declaration of Helsinki. All participants gave informed consent to take part in the study, which was approved by the Institutional Human Ethics Committee. These participants varied in age from 23 to 77 years (mean ± SD, 50.3 ± 19.0 years). Visual acuity (VA) ranged from -0.20 to 0.0 (0.00 ± 0.07 logMAR).

Exclusion criteria were: best-corrected logMAR VA of worse than 0.1 logMAR (VA was measured under standard testing conditions using a logMAR chart, retro illuminated to a luminance of 130 cdm^-2 ^[17] and each letter seen was scored as 0.02 log units, with guessing encouraged); retinal disease detected using a direct ophthalmoscope; glaucoma; lenticular opacities greater than grade 1 on the LOCS I grading scale [18]; prescribed medication associated with changes in retinal function.

The ARMD affected eyes were classified according to the International Classification and Grading System for Age-related Maculopathy (ARM) and Age-related Macular Degeneration (AMD) [19]. ARM refers to large soft drusen and pigmentary abnormalities of the retinal pigment epithelium (RPE) and the retina, whereas AMD refers to later stages of the disease such as GA, choroidal neovascularization, pigment epithelium detachment, and fibrous scaring of the macula [19]. Digital fundus photographs were taken using the Topcon non-mydriatic TRC-NW5S retinal camera (Topcon House, Bone Lane. Kennet Side, Newbury, Berkshire RG14 2PX, UK). Eyes were not dilated and one investigator classified all photographs.

The MMT was carried out on 17 ARM affected eyes of 17 participants aged from 55 to 82 (69.4 ± 7.7 years) and 12 AMD affected eyes of 12 participants aged from 65 to 78 (71.8 ± 4.3 years). VA ranged from -0.08 to 0.2 (0.04 ± 0.09 logMAR) for the ARM group, and from 0.2 to 0.76 (0.50 ± 0.21) for the AMD group. The eyes included did not have lenticular opacities greater than grade 1 on the LOCS I grading scale [18], and were not affected by any other ocular condition. The MMT scores from these groups were compared with those from 16 normal participants aged > 50 years, aged from 51 to 77 (67.1 ± 8.9 years).

### Materials

**Figure 1:**
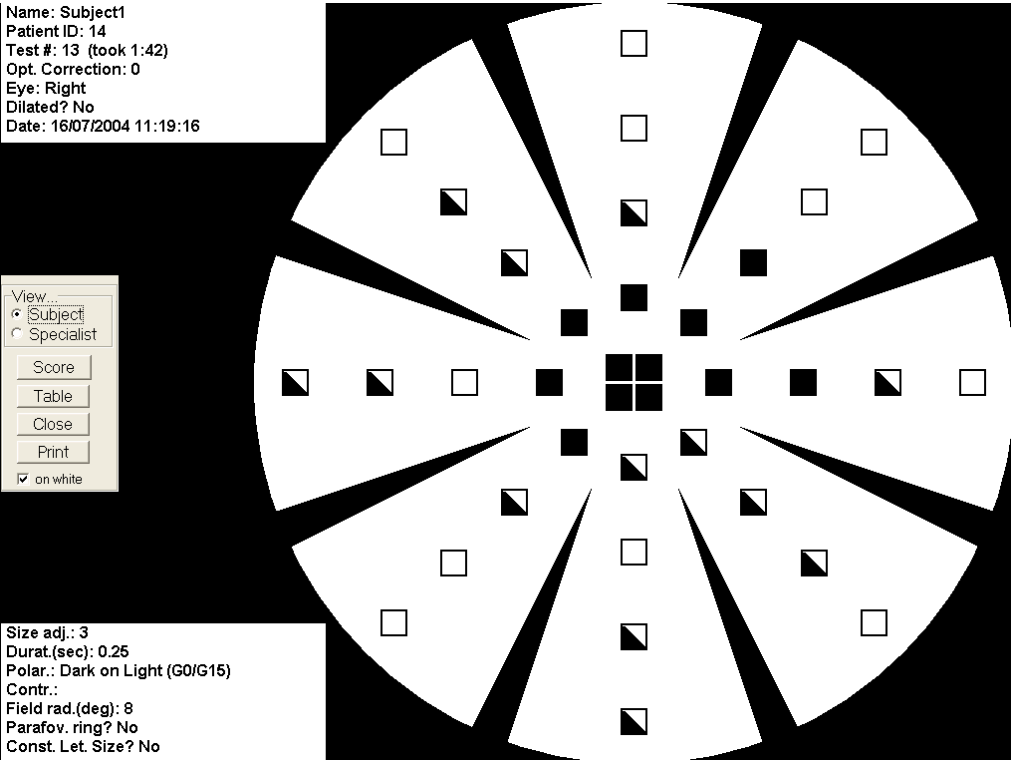
Subject view of the MMT results. Black squares indicate letters that were not seen, half-shaded squares indicate those that were seen but not correctly identified, and white squares indicate letters that were correctly identified.

The MMT is a software program used in conjunction with a desktop or laptop computer, designed specifically to map visual defects due to macular disease. The test screen displays a characteristic background pattern throughout the test, which resembled a 'wagon wheel'. Eight spokes point inwards, but do not reach the centre of the circular display area. This pattern provides the patient with sufficient peripheral landmarks to indicate the location of the centre of the circular display area [20], even if the centre is not directly visible to the patient (fig. 1).

When loaded for the first time, the program prompts the user to calibrate the system. It is necessary to measure the diameter of the wagon wheel, to the check the horizontal/vertical proportion of the monitor, and to set the monitor to maximum contrast and brightness. The diameter of the wagon wheel on the screen allows the system to suggest a correct viewing distance, such that the wagon wheel has the correct angular extent of 18° diameter. The viewing distance for our system was 76.2 cm.

The targets comprise of the Sloan letters [21] with a change in the width-to-height ratio from 5 × 5 to 4 × 5 for better legibility. Serifs are also added to the D to reduce the probability of confusion with O. Letter sizes vary according to eccentricity, starting at 5 pixels high, and increments of 5 pixels to 45 each. Thirty-two standard locations ensure that the letters do not 'collide' with the spokes of the wagon wheel. There are an additional four central locations.

### Procedure

All participants were seated comfortably, with their head supported using a chin rest, and the eye not being tested occluded. In order to ensure control of direction of gaze, each participant was asked, 'Do you have a sense of where the centre of the wagon wheel is?'. All participants responded positively, and were then instructed to direct their eye to the centre and to keep it there as still as possible. Participants with AMD were also advised that the centre of the wagon wheel may seem to disappear. As the background illuminance was low (6 lux), and our participants did not have lens opacities greater than grade one of the LOCS I scale, we were not concerned about glare, and therefore selected the black letters on a white background presentation mode. The following explanation of the test was given to every participant; 'Black letters will appear one at a time anywhere on the white wagon wheel. It is important that you do not move your eye to try and look at them. When you see a letter, say the name of it out loud. If you are not sure what the letter is, have a guess'.

The same testing room was used for each test with background lighting switched off. When both eyes matched the inclusion criteria, the right eye was tested, and when only one eye was suitable for inclusion, this eye was tested. The prescription providing best-corrected visual acuity at the test distance was placed into a trial frame, with the eye not being tested occluded. Full aperture trial lenses were used.

When each participant was ready the examiner pressed the mouse button and a letter appeared in an unpredictable location in the visual field, remaining visible for 234 msec. The participant told the examiner which letter they perceived and the examiner entered this response into the computer for scoring. This prompts display of the next letter, and so on. There are three categories of response; 1) target not detected, 2) target detected, but not recognized (including incorrect responses), 3) target correctly recognized. The final score is calculated using the following system; target not detected = 0, target detected but not recognized = 1, target detected = 2. The score is displayed at the end of the test and the participant is able to view a 'map' of the areas seen and unseen.

Data were collected by two optometrists, HB and LD, during two sessions separated by one hour from the same subjects. A trial run was completed for each subject by HB in the first session and LD in the second session to encourage test familiarity and reduce learning effects. In session 1 the first experimental test was carried out by HB, and the second by LD, and in session two this order was reversed The study was designed to assess reproducibility (HB1-LD1, LD2-HB2), and repeatability (HB1-HB2, LD1-LD2),

Data analysis employed the independent-samples *t*-test for comparing ages and the chi-squared test to compare proportion of males by group. The MMT scores are non-continuous data, and so we used nonparametric statistical tests in our analysis. The Mann-Whitney U test was used to compare MMT scores between groups. When assessing the effect of ARMD on MMT score, power analysis shows that the group sizes were sufficient to have an 80% chance of detecting a difference in means of 5 MMT points at the 5% level of significance using the independent samples t-test.

## Results

### Reliability

**Figure 2:**
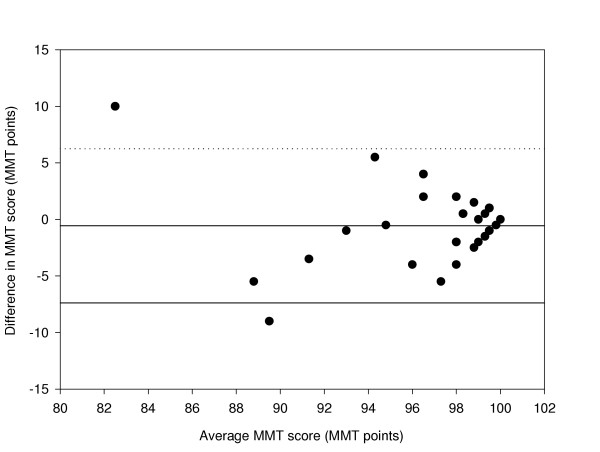
Difference in MMT score between HB1 and HB2 compared with the mean (n = 31 eyes). The mean bias is represented by the solid line and the 95 % confidence limits are represented by the dotted lines.

**Figure 3:**
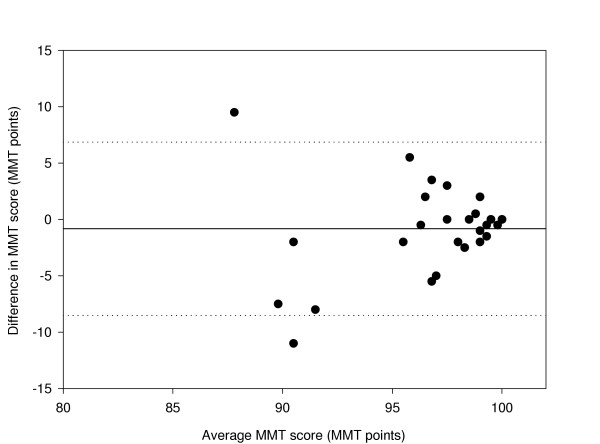
Difference in MMT score between HB1 and LD1 compared with the mean (n = 31 eyes). The mean bias is represented by the solid line and the 95 % confidence limits are represented by the dotted lines.

**Figure 4:**
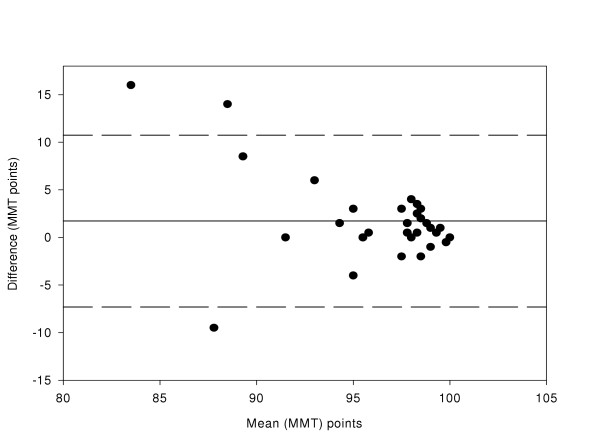
Difference in Macular Mapping Test (MMT) value between LD1 and LD2, compared with the mean (N = 30 eyes). The mean bias is represented by the solid line, and the 95% confidence limits are known by the dashed lines.

**Figure 5:**
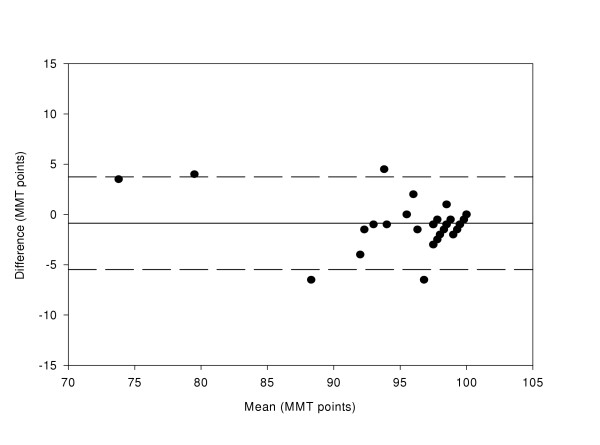
Difference in Macular Mapping Test (MMT) value between LD2 and HB2, compared with the mean (N = 30 eyes). The mean bias is represented by the solid line, and the 95% confidence limits are known by the dashed lines.

Average MMT scores ranged from 85.5 to 100.0 MMT points. Reproducibility was determined by comparing HB1-LD1 and LD2-HB2, and repeatability was determined by comparing HB1-HB2 and LD1-LD2. The differences between data sets for two of the comparisons (HB1-HB2 and HB1-LD1) were normally distributed and these were used for analysis. See figures 2 to 5 for a graphical representation of test-retest data of the HB1-HB2, HB1-LD1, LD1-LD2, and LD2-HB2 comparisons respectively.

Accurate analysis of test-retest data can be achieved using the coefficient of repeatability [22,23]. This gives the 95% confidence limits for the amount of difference between two sets of results. It is calculated as 1.96 multiplied by the standard deviation of the mean differences between the two sets of data. The coefficient of repeatability is ± 6.70 for the HB1-HB2 comparison, ± 7.24 for the HB1-LD1 comparison, ± 7.4 for the LD1-LD2 comparison, and ± 4.3 for the LD2-HB2 comparison.

**Figure 6:**
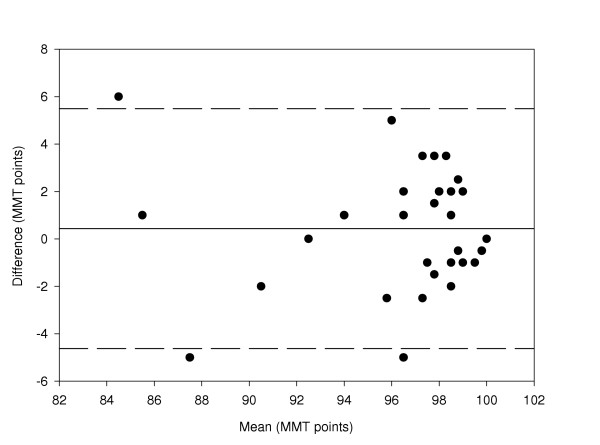
Difference in Macular Mapping Test (MMT) value between HB1 and LD2, compared with the mean (N = 30 eyes). The mean bias is represented by the solid line, and the 95% confidence limits are known by the dashed lines.

A comparison between HB1 and LD2 has also been made. The coefficient of repeatability is ± 4.9 and the data is shown in figure 6.

### Effect of ARM

Of the ARM participants, 12 had soft distinct drusen, three had soft distinct drusen with hyperpigmentation of the RPE, and two had soft indistinct drusen. The ARM group and the subset of nomals were matched for age (t = -0.783; p = 0.439) and gender [χ^2 ^(1) = 0.501, p = 0.579]. Scores ranged from 85.5 to 100.0 for the subset of normals and from 79.0 to 99.0 for the ARM group. The mean MMT score was 94.5 points (94.6 ± 4.8) for the over 50 normal group and 95.0 points (94.9 ± 4.7) for the ARM group, and there was no significant difference between the groups (z = -0.216, p = 0.829). Power analysis shows that the group sizes were sufficient to have an 80% chance of detecting a difference in means of 5 MMT points at the 5% level of significance using the unpaired t-test.

### Effect of AMD

All AMD participants had GA, characterised by any sharply delineated roughly round or oval area of hypopigmentation or depigmentation, or apparent absence of the RPE, in which choroidal vessels were more visible than in surrounding areas. The size of the lesion was at least 1/8 disc diameters in size. The AMD group and the subset of normals were matched for age (t = -1.613, p = 0.119) and gender [χ^2 ^(1) = 0.619, p = 0.821). Scores ranged from 9.0 to 92.0 for the AMD group. The mean MMT score was 62.0 points (61.9 ± 23.8) for the AMD group. The difference in scores between the over 50 and the AMD group is statistically significant (z = 3.761, p < 0.001).

**Figure 7:**
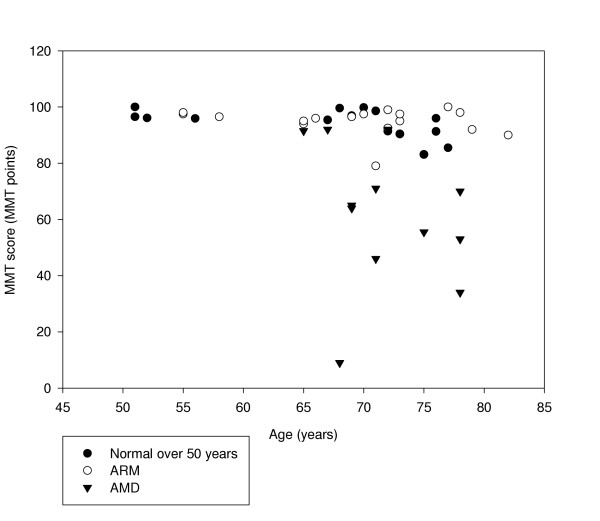
First MMT scores taken from normal (over 50 years), ARM and AMD participants plotted against age.

Figure 7 shows the effect of age on MMT score for the normal, ARM and AMD groups.

## Discussion

The primary objective of this study was to determine the reliability of the MMT. The developers of the MMT assessed its reliability by performing the test twice on 20 eyes. The conditions were made harder between the first and second run by decreasing the size of the letters by 5 pixels, and 92 % of all tested locations in the participants behaved within expectation. It was concluded that the test procedure reflects the functional topography with reasonable accuracy and reliability [24]. Our reliability data has been used to determine the decrease in MMT score that is needed to indicate a clinical change between tests. The MMT scores are repeatable to within ± 7.0 points, indicating that the MMT score has to change by more than 14 points for the change to be clinically significant. This value could be useful if MMT score was being used as an outcome measure for monitoring progression of ARMD, or for monitoring the effect of an intervention, e.g. a nutritional supplement.

A limitation of this study design is that the order of the assessor in each of the two sessions was not randomly assigned. This would have permitted assessment of the variance components, assessor, order, and session. However, a comparison has been made between HB1 and LD2, and the coefficient of repeatability was smaller than for other comparisons. This, therefore, does not affect the reliability conclusions draw.

Comparisons between ARM-affected eyes and age- and gender-matched controls yielded no significant difference, although this part of the study was powered to detect a difference between groups. There was significant reduction in MMT score between AMD-affected eyes and age- and gender-matched controls. Our results show that the MMT is not suitable for screening of ARM.

## Conclusion

Although the reliability data indicates variability, the MMT has the advantage over, for example, the Amsler grid that it uses a letter target, has a peripheral fixation aid, and it provides a numerical score. The score could be beneficial in clinic and home monitoring of AMD progression, as well as provide an outcome measure in clinical research.

## Competing interests

The author(s) declare that they have no competing interests.

## Authors' contributions

HB participated in the design of the study, the acquisition, analysis, and interpretation of the data, and drafting of the final manuscript. LD participated in the design of the study, aquisitiion of data, and the final drafting of the manuscript. FE participated in the design of the study and the final drafting of the manuscript. All authors read and approved the final manuscript.

## Pre-publication history

The pre-publication history for this paper can be accessed here:
